# Microtubule Depolymerization by Kinase Inhibitors: Unexpected Findings of Dual Inhibitors

**DOI:** 10.3390/ijms18122508

**Published:** 2017-11-23

**Authors:** Kenji Tanabe

**Affiliations:** Medical Research Institute, Tokyo Women’s Medical University, Tokyo 162-8666, Japan; tanabe.kenji@twmu.ac.jp; Tel.: +81-3-5269-7487

**Keywords:** microtubules, kinase inhibitor, dual inhibitor, mitotic arrest, intracellular traffic

## Abstract

Microtubule-targeting agents are widely used as clinical drugs in the treatment of cancer. However, some kinase inhibitors can also disrupt microtubule organization by directly binding to tubulin. These unexpected effects may result in a plethora of harmful events and/or a misinterpretation of the experimental results. Thus, further studies are needed to understand these dual inhibitors. In this review, I discuss the roles of dual inhibitors of kinase activity and microtubule function as well as describe the properties underlining their dual roles. Since both kinase and microtubule inhibitors cause cell toxicity and cell cycle arrest, it is difficult to determine which inhibitor is responsible for each phenotype. A discrimination of cell cycle arrest at G0/G1 or G2/M and/or image analyses of cellular phenotype may eventually lead to new insights on drug duality. Because of the indispensable roles of microtubules in mitosis and vesicle transport, I propose a simple and easy method to identify microtubule depolymerizing compounds.

## 1. Introduction

Microtubules play indispensable roles in various cellular processes such as chromosomal segregation during mitosis and vesicle trafficking during endocytosis [1]. The roles of microtubules in mitosis indicate that agents affecting microtubule function can be effective anti-cancer drugs [2,3], which has led to the development of numerous natural and synthetic small molecule compounds [3]. However, these microtubule-targeting agents display huge variations in terms of their chemical structures and tubulin-binding sites [3,4,5], indicating that they may also target proteins other than tubulin. On the other hand, cellular kinases play important roles in cell growth and differentiation, and many kinase inhibitors have been developed as anti-cancer drugs [6]. Several of these have been found to also affect microtubule function. For example, tivantinib is a c-met inhibitor that depolymerizes microtubules [7,8]. These findings are important because they can alter the interpretation of key experimental results, particularly when evaluating the unfavorable side effects of drugs in clinical use. In this review, I discuss several compounds that inhibit kinase activity and affect microtubule function; these compounds are listed in Table 1. I also propose a simple and easy method to identify microtubule-depolymerizing compounds.

## 2. A Clinically Tested c-Met Kinase Inhibitor Is Also a Microtubule Inhibitor

The hepatocyte growth factor (HGF) receptor, c-met, frequently shows sustained activation by its mutation or overexpression in cancer cells [24,25]. Tivantinib was initially developed as a non-ATP competitive inhibitor of c-met and subsequently found to inhibit the proliferation of cells expressing wild-type c-met or the constitutively active c-met mutant [26]. Likewise, c-met knockdown by RNAi inhibited cell proliferation. Due to its effects on cell proliferation, this compound entered a clinical trial. However, subsequent experiments showed that tivantinib also inhibits other proteins, which led to changes in the way experimental results are interpreted. For example, Basillico et al. reported that tivantinib induces cell death regardless of c-met expression [7], whereas Katayama et al. showed that tivantinib, but not other c-met inhibitors, reduces the viability of c-met-addicted as well as non-addicted cells [8]. Other c-met inhibitors, such as crizotinib and PHA-665752, arrest the cell cycle at G0/G1, but not at G2/M. By contrast, tivantinib cannot affect the transition from G0/G1; instead, it arrests the cell cycle at G2/M, a hallmark of microtubule disruption. COMPARE analysis, which involves the in silico screening of a database of drugs affecting 39 cancer cell lines (JFCR39), helped to identify compounds that depolymerize microtubules in vitro [8]. Tivantinib inhibits microtubule polymerization by directly binding to tubulin via the colchicine-binding site [27].

Although tivantinib is an inhibitor of c-met activity and microtubule function, the results of early studies were difficult to interpret because other c-met inhibitors, as well as c-met knockdown, induced similar phenotypes (i.e., inhibited cell proliferation and/or induced cell death). Further studies, however, showed that tivantinib depolymerized microtubules, resulting in cell cycle arrest at G2/M, whereas other c-met inhibitors and c-met knockdown caused cell cycle arrest at G0/G1 [8].

## 3. Examples of Kinase Inhibitors That Disrupt Microtubule Function

In this section, I discuss compounds that were developed as kinase inhibitors but subsequently found to depolymerize microtubules. These dual roles are due to the fact that kinase inhibitors are usually tested for specificity using a kinase panel [28,29] but not screened for their possible effects on other proteins.

IC261, a casein kinase 1 delta and epsilon (CK1δ/ε) inhibitor [30], induces mitotic arrest in cells. CK1δ plays roles in mitosis and localizes to mitotic spindles [31], indicating that IC261 is an effective anti-cancer drug [32]. PF670 is another CK1δ/ε inhibitor; however, it is structurally different from IC261 [9]. PF670 inhibits Wnt, a downstream effector of CK1δ/ε, but it has no effects on cell survival. By contrast, IC261 induces cell death, even at low concentrations, which fails to inhibit CK1 and Wnt. IC261 binds to the colchicine-binding site of tubulin and inhibits microtubule function [9].

CMPD1 is a substrate-selective inhibitor of p38α [33] that inhibits the p38-dependent phosphorylation of mitogen-activated protein kinase (MAPK)-activated protein kinase 2 (MK2), a protein within one of the checkpoint pathways, in response to DNA damage. Therefore, the inhibition of MK2 by CMPD1 represents a good anti-cancer approach. Subsequent studies showed that another MK2 inhibitor, MK2i [34], and MK2 knockdown cannot induce cell death [10]. Furthermore, low concentrations of CMPD1 that cannot inhibit MK2 activity can still arrest the cell cycle at G2/M and reduce cell survival. Thus, it was concluded that mitotic arrest and apoptosis induced by CMPD1 is independent of MK2 inhibition; instead, it is due to microtubule depolymerization [10].

S9 is an inhibitor of phosphoinositide 3-kinase (PI3K) and mammalian target of rapamycin (mTOR), and it inhibits the PI3K-Akt pathway [11]. High concentrations of S9 arrest the cell cycle at G0/G1, similar to other PI3K-Akt inhibitors, whereas low concentrations of S9 arrest the cell cycle at G2/M but not at G0/G1. S9 binds to the colchicine-binding site within tubulin, inhibits microtubule polymerization, and disrupts the microtubule structure.

LIM kinase (LIMK) regulates the actin cytoskeleton by phosphorylating cofilin, and several lead compounds have been developed as LIMK inhibitors, two of which can induce cell toxicity in vivo [12]. Chemical modification of these two lead compounds led to the development of two derivatives that either inhibit LIMK or induce cell toxicity, suggesting that LIMK is not important for cell survival. Further experiments revealed that two lead compounds induces cell toxicity by direct inhibition of microtubules [12].

Fascaplysin, a marine natural product, is an inhibitor of cyclin-dependent kinase 4 (Cdk4) that regulates the G0/G1 checkpoint [35,36]. This compound is highly toxic to cells because of its DNA-intercalating activity. Although *N*-(biphenyl-2-yl) tryptoline (BPT) was developed as its analogue [13], it cannot bind DNA, yet it can inhibit the Cdk4-cyclinD complex. BPT also arrests the cell cycle at G2/M, a hallmark of microtubule inhibition, and induces microtubule depolymerization. The authors concluded that BPT is a unique anti-cancer drug that can arrest the cell cycle at G0/G1 by inhibiting Cdk4 and at G2/M by disrupting microtubules [13]. 

BKM120 (buparlisib) is one of most clinically advanced PI3K inhibitors [37,38,39]. Cytotoxic profiles of BKM120 to various cell lines are distinct from those of GDC-0941, another PI3K inhibitor [14]. Furthermore, cells whose growth is independent of PI3K as well as the cells expressing dominant-active Akt (a downstream effector of PI3K) are resistant to GDC-0941, but sensitive to BKM120. Thus, the cytotoxic effect of BKM120 appears to be independent of PI3K. In fact, BKM120 at high concentration induces mitotic arrest at G2/M, irrespective of PI3K levels, and blocked microtubule polymerization in vitro. Recently, two derivatives of BKM120 were chemically synthesized, and each derivative possesses either PI3K inhibitory activity or microtubule depolymerizing activity [40].

## 4. Image Analyses of Cellular Phenotype That Led to the Identification of Dual Inhibitors of Kinases and Microtubules

Rigosertib was originally identified as polo-like kinase 1 (Plk1) inhibitor [41] and is now on phase 3 clinical trial. However, this compound causes mitotic arrest and apoptosis of cells, a phenotype resembling that of microtubule depolymerizing agents not that of Plk1 siRNA or another Plk1 inhibitor, BI2536 [15]. Although there were several controversial reports about the microtubule depolymerizing activity of rigosertib [41,42,43], high content image analysis classified rigosertib as a microtubule depolymerizing agent [44]. Recent CRISPR-based chemical genetic screens also showed that rigosertib is a microtubule depolymerizing agent, and the direct binding of the compound to tubulin is required for its cytotoxicity [45].

CAS 879127-08, a specific inhibitor of epidermal growth factor receptor (EGFR) [46,47], is highly toxic to cells, and it induces cancer cell apoptosis [48]. In my previous study [16], cells were treated with inhibitors of the EGFR pathway (14 compounds, including CAS 879127-08 and nocodazole), and the acquired cell images were analyzed. I found that CAS 879127-08 was not in the cluster of other EGFR inhibitors; instead, it was identified in the cluster of nocodazole. The intracellular trafficking of transferrin was affected in CAS 879127-08-treated cells, which was indicative of microtubule disruption. In subsequent biochemical and biological assays, CAS 879127-08 directly inhibited microtubule polymerization [16,49]. Thus, in some cases, image analyses may help in the identification of target-specific compounds.

## 5. Examples of Microtubule Disrupting Agents That Also Inhibit Kinases

The screening of anti-cancer agents led to the identification of 3-substituted 7-phenylpyrrolo[3,2-f]quinolin-9(6*H*)-one [17]. This compound arrests the cell cycle at G2/M and inhibits microtubule polymerization. Its chemical structure resembles some inhibitors of the PI3K-Akt pathway, and this compound inhibits proteins within this pathway. Subsequently, a panel of 386 kinases was surveyed, and 3-substituted 7-phenylpyrrolo[3,2-f]quinolin-9(6*H*)-one was found to target several kinases, including aurora kinase, FMS-like tyrosine kinase 3 (FLT3), glycogen synthase kinase 3α (GSK3α), MAPK/extracellular signal-regulated kinase (ERK) kinase (MEK), and JUN amino-terminal kinase (JAK2) [17]. The screening of anti-cancer agents also led to the identification of 5,7-dibromo-*N*-(*p*-thiocyanomethylbenzyl) isatin (KS99), an apoptosis-inducing [50] and microtubule depolymerizing compound that affects Akt phosphorylation by directly inhibiting Bruton’s tyrosine kinase [18].

Two distinct thiazolidinone derivatives that depolymerize microtubules and induce cell cycle arrest at G2/M were developed [19]. However, their effects on cell survival were different; cells treated with probe 1 died, whereas those treated with probe 2 survived. Using the KINOMEscan platform [51,52], dual specificity tyrosine-phosphorylation-regulated kinase (DyrK), a serine/threonine kinase mediating cell survival in response to microtubule damage [53], was identified as a target of probe 1, but not of probe 2, indicating that probe 1 disrupts microtubules and inhibits DyrK. The authors concluded that this dual role of probe 1, an inhibitor of tubulin polymerization and DyrK kinase, represents a novel anti-cancer strategy [19].

Nocodazole, a well-studied microtubule depolymerizing agent, binds to several kinases in vitro [54] and perturbs the MAPK pathway in vivo [16,55]. It is possible to design an inhibitor with a dual role in the inhibition of kinase activity and microtubule function. Biarylaminoquinazolines were developed using information from a structural similarity search of tyrosine kinase inhibitors and microtubule depolymerizing agents [20,56].

## 6. Disruption of Microtubules by Non-Kinase Inhibitors

Dual inhibitors can be found in non-kinase inhibitors. SB225002 was initially developed as an antagonist of the G-protein coupled receptor, C-X-C motif chemokine receptor 2 (CXCR2) [57], but later found to exhibit cytotoxicity and to arrest the cell cycle at G2/M. This compound affects microtubules in mitotic cells by binding to the vinblastine-binding site within tubulin [21]. On the other hand, rotenone is a mitochondrial complex I inhibitor, whose inhibition of cellular microtubules was described five decades ago [58,59]. It binds to the colchicine-binding site within tubulin [22], thus disorganizing microtubules and inducing cell cycle arrest. Tyrosinase inhibitors are synthetic derivatives of chalcones and pyrazolines [23]. They induce microtubule depolymerization and exhibit cytotoxicity in vitro. 

## 7. A Simple Method to Screen Agents for Microtubule Disruption

**Figure 1:**
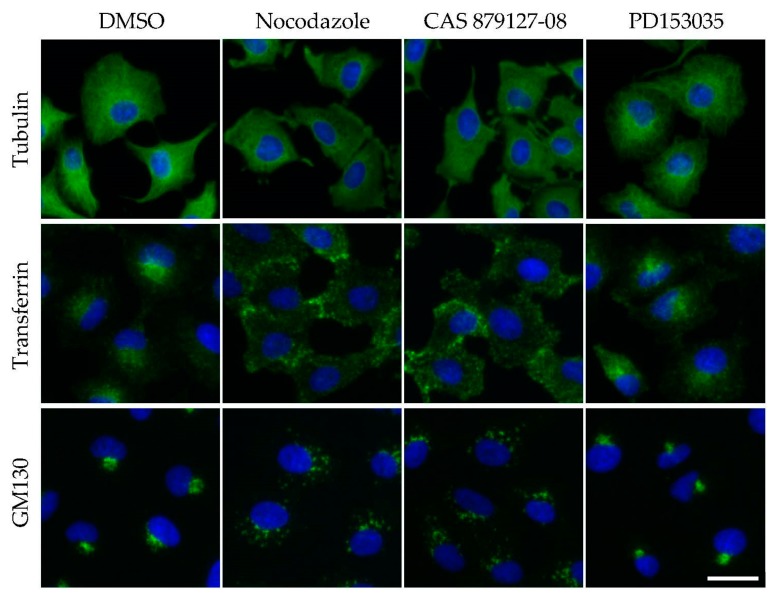
Microtubule depolymerization impairs intracellular trafficking. A549 cells were treated with DMSO (0.1%), nocodazole (1 µM), CAS 879127-08 (1 µM), or PD153035 (epidermal growth factor receptor (EGFR) kinase inhibitor, 1 µM) for 1 h. Cells were fixed and immunofluorescently stained to visualize microtubules using an anti-tubulin antibody (Upper panels, green) or Golgi structures using an anti-GM130 antibody (Middle panels, green). To visualize internalized transferrin, cells were incubated with fluorescently labeled transferrin for 5 min (Bottom panels, green). Cells were counterstained with Hoechst33342 (blue). Scale bar, 20 µm.

It is a challenge to predict whether a given compound possesses microtubule depolymerizing activity [5], because microtubules can be inhibited via direct and indirect mechanisms in vivo [3]. Biochemical assays using purified tubulin have been instrumental in defining direct mechanisms [60,61], whereas assays that assess cytotoxicity after treatment are good indicators of microtubule disruption, although cytotoxicity can be caused by other cellular defects. For example, the effects of tivantinib and CAS 879127-08 on microtubules were not understood for a long time, because the inhibition of these targets (c-met and EGFR) also inhibited cell proliferation. Here, I propose an easy and simple approach to determine whether a given compound can disrupt microtubule function (Figure 1).

Although most of the kinase inhibitors listed in Table 1 induce mitotic arrest, CAS 879127-08 impairs transferrin internalization, indicating that the intracellular trafficking is a good indicator of microtubule disruption [62,63]. For example, microtubules and their motor proteins, kinesin and dynein, mediate the transport of intracellular vesicles and organelles throughout cells. Indeed, a disruption of microtubule organization impairs the transport of these organelles. Microtubule disruption by nocodazole or CAS 879127-08 affects the trafficking of vesicles from the plasma membrane to early endosomes, whereas the Golgi apparatus becomes fragmented (Figure 1). To study endocytosis, cells were incubated with fluorescently labeled transferrin for 5–30 min, fixed, and then observed under a microscope [16]. Transferrin-containing vesicles were transported to the perinuclear region in control cells, but remained at the cell periphery in microtubule-disrupted cells. To evaluate the integrity of the Golgi apparatus, fixed cells were fluorescently immunostained [64] or fluorescently labeled with wheat germ agglutinin [65]. Thus, this simple method can be used to screen the effects of a library of compounds on microtubule function.

## 8. Perspectives

In this review, I discussed several kinase inhibitors that were found to possess microtubule depolymerizing activity, as well as microtubule-targeting drugs that were found to inhibit kinases.

The following three caveats have to be kept in mind when considering dual inhibitors. The first is to know the exact mechanisms of drug action. Microtubule depolymerizing and polymerizing agents cause mitotic arrest followed by apoptosis, and this toxic effect is more apparent in cancer cells than normal cells. In fact, several microtubule inhibitors are in standard clinical use. On the other hand, many kinase inhibitors that affect cell cycle progression have been developed but fail to achieve results in the clinic as compared to microtubule inhibitors. Tivantinib is a rather rare case that was initially developed as a kinase inhibitor and is still useful in clinical trial. Only after years of use in the clinic, however, was tivantinib found to exert microtubule-depolymerizing activity as well as anti-kinase activity [7,8]. Thus, once a cell growth inhibitory effect of a kinase inhibitor is detected, a hidden target, such as microtubules, might be easily overlooked. In addition, the protein that is targeted by a drug is likely to determine which type of cells are targeted by the drug. For example, tivantinib as a c-met inhibitor is expected to be toxic to the c-met-addicted cells, whereas the same drug as a microtubule inhibitor would be toxic to the c-met-addicted and non-addicted cells. In any case, it is important to consider the possibility of dual activity, since misunderstanding the mechanism of action might mislead therapy strategies, particularly in future personalized medicine. Secondly, when one compound targets multiple distinct proteins, these inhibitory properties may vary from one target to another. It emerges as an effective drug concentration and/or an effective drug exposure time that is required to induce a target-dependent phenotype. For example, in the case of a dual inhibitor, BKM120, the concentration necessary to induce mitotic arrest as a microtubule depolymerizing agent is 5- to 10-fold higher to that necessary to inhibit PI3K activity [14]. To recognize a target-specific concentration window would be vital to ensure the correct usage of inhibitor. A third concern on a dual inhibitor is the deconvolution of dual activity into two chemical derivatives that possesses only kinase-inhibiting activity or only microtubule depolymerizing activity. This approach was reported for BKM120 and tubulin/DyrK inhibitor [19,40] and might be useful for developing next-generation drugs.

I recently reported that CAS 879127-08, an EGFR inhibitor, can inhibit EGFR and disrupt microtubules [16]. In this previous report, high content analyses of acquired cell images were instrumental in arriving at the above conclusion, whereas, in the present review, I have proposed a simple detection method of microtubule depolymerization. In any case, the inhibition of two targets by a single agent may have added benefits in the treatment of cancer for two reasons. Firstly, the inhibition of two independent pathways enhances drug efficacy [13,19]. Secondly, the likelihood of drug-tolerant cells arising is low, because these cells have to find a means to escape inhibition by two independent pathways. Thus, further research employing, for example, high content analyses of images coupled with a detection method for microtubule inhibitors is needed to develop more effective dual inhibitors.

## Figures and Tables

**Table 1:** List of dual inhibitors of microtubules and other target.

Group	Compound	Target	Order ^1^	Evidence ^2^	Phenoytpe ^3^
Kinase inhibitor	Tivantiniv [7,8]	c-met	Kinase → MT	Other inhibitor	M
	IC261 [9]	CK1	Kinase → MT	Other inhibitor	M
	CMPD1 [10]	MK2	Kinase → MT	Other inhibitor	M
	S9 [11]	Akt	Kinase → MT	Other inhibitor	M
	LIMK inhibitor [12]	LIMK	Kinase → MT	Other inhibitor	M
	BPT [13]	Cdk4	Kinase → MT	Known function	M
	BKM120 [14]	PI3K	Kinase → MT	Other inhibitor	M
	Rigosertib [15]	Plk1	Kinase → MT	Other inhibitor	M
	CAS 879127-08 [16]	EGFR	Kinase → MT	Other inhibitor	T
	3-substituted 7-Phenylpyrrolo [3,2-f]quinolin-9(6H)-ones [17]	multi-kinase	MT → Kinase	Akt inactivation	M
	KS99 [18]	Btk	MT → Kinase	Akt inactivation	M
	Tubulin/DyrK inhibitor [19]	DyrK	MT → Kinase	Other inhibitor	M
	Biarylaminoquinazolines [20]	Tyr. kinase	Predesigned	Predesigned	M
Non-kinase inhibitor	SB225002 [21]	CXCR2	GPCR → MT	Other inhibitor	M
	Rotenone [22]	Mitochondria	Mito. → MT	Concentration	M
	Tyrosinase inhibitor [23]	Tyrosinase	Tyrosinase → MT	Other inhibitor	M

^1^ Order of target discovery; ^2^ Evidence leading to the discovery of a second target; ^3^ Observed cellular phenotype (M: mitotic arrest; T: unusual traffic). MT, microtubules; CK1, casein kinase 1; MK2, mitogen-activated protein kinase (MAPK)-activated protein kinase 2; LIMK, LIM kinase; BPT, *N*-(biphenyl-2-yl) tryptoline; Cdk4, cyclin-dependent kinase 4; BKM120, buparlisib; PI3K, phosphoinositide 3-kinase; Plk1, polo-like kinase 1; EGFR, epidermal growth factor receptor; KS99, 5,7-dibromo-*N*-(*p*-thiocyanomethylbenzyl) isatin; Btk, Bruton’s tyrosine kinase; DyrK, dual specificity tyrosine-phosphorylation-regulated kinase; CXCR2, C-X-C motif chemokine receptor 2.

## Abbreviations

BPT	*N*-(biphenyl-2-yl) tryptoline
Btk	Bruton’s tyrosine kinase
Cdk4	cyclin-dependent kinase 4
CK1	casein kinase-1
CXCR2	C-X-C motif chemokine receptor 2
DyrK	dual specificity tyrosine-phosphorylation-regulated kinase
EGFR	epidermal growth factor receptor
ERK	extracellular signal-regulated kinase
FLT3	FMS-like tyrosine kinase 3
GSK3α	glycogen synthase kinase 3α
HGF	hepatocyte growth factor
JAK2	JUN amino-terminal kinase
KS99	5,7-dibromo-*N*-(*p*-thiocyanomethylbenzyl) isatin
LIMK	LIM kinase
MAPK	mitogen-activated protein kinase
MEK	MAPK/ERK kinase
MK2	MAPK-activated protein kinase 2
MT	microtubules
mTOR	mammalian target of rapamycin
PI3K	phosphoinositide 3-kinase
Plk1	polo-like kinase 1
